# Case Report: Delayed epirubicin extravasation-induced ulceration in a dog managed without surgical intervention using adjunctive hyperbaric gaseous cryotherapy

**DOI:** 10.3389/fvets.2026.1939525

**Published:** 2026-10-28

**Authors:** Chaeyeon Gloria Kim, Ji-Hwan Park, Soon-Wuk Jeong

**Affiliations:** 1 Department of Veterinary Surgery, College of Veterinary Medicine, Konkuk University, Seoul, Republic of Korea; 2 Leaders Animal Medical Center, Seongnam, Republic of Korea

**Keywords:** anthracycline, chemotherapy complication, conservative management, delayed presentation, epirubicin, extravasation, hyperbaric gaseous cryotherapy, wound healing

## Abstract

Extravasation of anthracyclines can result in progressive tissue injury, ulceration, and necrosis, and may ultimately require surgical intervention. Management of delayed presentations remains challenging, as recommended treatments, including dexrazoxane, dimethyl sulfoxide (DMSO), and cold compresses, are most effective when initiated promptly after the extravasation event. A 9-year-old spayed female Poodle with suspected stage IV multicentric lymphoma developed a progressive ulcerative lesion 17 days after suspected epirubicin extravasation, by which time the recommended treatment window for standard interventions had elapsed. Given the progressive ulceration, severity of tissue injury, and delayed presentation, surgical debridement was considered. However, conservative management was pursued owing to the patient’s limited prognosis and the owner’s preference to prioritize quality of life. Treatment included hyperbaric gaseous cryotherapy (HGC) as the principal adjunctive modality, based on prior clinical experience in wound management and pain control. Dexrazoxane, topical DMSO, and ice-pack application were also incorporated despite the elapsed therapeutic window, as few alternative treatment options remained at that stage. Progressive wound healing was observed over the course of treatment, with complete re-epithelialization achieved by day 93 without surgical intervention. The wound remained fully healed until the patient died of progressive lymphoma on day 138. This case documents complete wound resolution without surgery in a dog with delayed epirubicin extravasation managed with multimodal conservative treatment incorporating adjunctive HGC.

## 1 Introduction

Extravasation of intravenously administered chemotherapeutic agents is a recognized complication of cancer treatment, with vesicant drugs posing the greatest risk of tissue injury (1, 2). Anthracyclines, including doxorubicin and epirubicin, are among the most clinically significant vesicants, as their extravasation can lead to progressive tissue injury, ulceration, and necrosis that may ultimately require surgical intervention (1, 2). The severity of extravasation-associated lesions varies according to drug classification, administered concentration and volume, and the anatomical site of injury (3).

Epirubicin is an anthracycline analogue of doxorubicin developed through epimerization to reduce cardiotoxicity while preserving antitumor activity (4–6). In veterinary oncology, epirubicin has been used in the treatment of various canine malignancies, including lymphoma, hemangiosarcoma, and histiocytic sarcoma (7–9). In canine lymphoma, it has been used as an alternative to doxorubicin in multi-agent chemotherapy protocols (7). Despite its improved cardiac safety profile, the vesicant properties of epirubicin remain a clinical concern.

Recommended treatments for anthracycline extravasation include dexrazoxane, topical dimethyl sulfoxide (DMSO), and cold compresses, all of which are most effective when initiated promptly after the extravasation event (1, 10). Other approaches described in veterinary patients include the modified Villalobos technique, involving multiple small incisions and local saline infiltration following doxorubicin extravasation (11). Dexrazoxane is the only U.S. Food and Drug Administration (FDA)-approved agent for anthracycline extravasation in human medicine (12). However, its clinical use may be limited by the need for early administration, immediate availability, and cost (1, 13, 14). Despite the clinical significance of chemotherapy extravasation, veterinary evidence remains limited to retrospective studies, small case series, and individual case reports (3, 11, 15). Published evidence regarding the management of delayed presentations remains limited. Treatment of delayed presentations becomes challenging once progressive ulceration and necrosis develop, as surgical debridement and reconstructive procedures may be required (15, 16).

This report describes the conservative management of delayed epirubicin extravasation-associated ulceration in a dog with advanced-stage lymphoma, in a presentation where surgical management would typically be considered. Adjunctive hyperbaric gaseous cryotherapy (HGC), which delivers high-pressure carbon dioxide (CO₂) gas to affected tissues, was incorporated into the conservative treatment strategy. To the authors’ knowledge, this represents the first veterinary report of complete wound resolution achieved through conservative management incorporating adjunctive HGC in such a delayed and severe presentation of anthracycline extravasation.

## 2 Case presentation

A 9-year-old spayed female Poodle was diagnosed with stage IV multicentric lymphoma based on generalized peripheral lymphadenopathy and fine-needle aspiration cytology of an enlarged submandibular lymph node. Abdominal ultrasonography revealed diffuse, heterogeneous splenic echogenicity with a honeycomb-like pattern, suggestive of splenic involvement. Although splenic involvement was not cytologically confirmed, the disease was clinically considered stage IV and, in the absence of systemic clinical signs, was classified as substage a. A modified L-CHOP chemotherapy protocol, consisting of L-asparaginase, cyclophosphamide, epirubicin, vincristine, and prednisolone, was initiated. Epirubicin was selected as the anthracycline component of the protocol in consideration of its reported lower cardiotoxicity compared with doxorubicin (4–6, 8, 17).

Epirubicin hydrochloride 30 mg/m^2^ (Boryung Corporation, Seoul, South Korea) was diluted in 0.9% normal saline to a total volume of 14.4 mL and administered manually over 20 min. A 24-gauge catheter was placed in the left cephalic vein on the first attempt immediately prior to administration. The catheter was assessed every minute throughout administration by flushing with saline and confirming blood return. After approximately one-third of the epirubicin had been administered, mild swelling was noted proximal to the catheter site (Figure 1).

**Figure 1:**
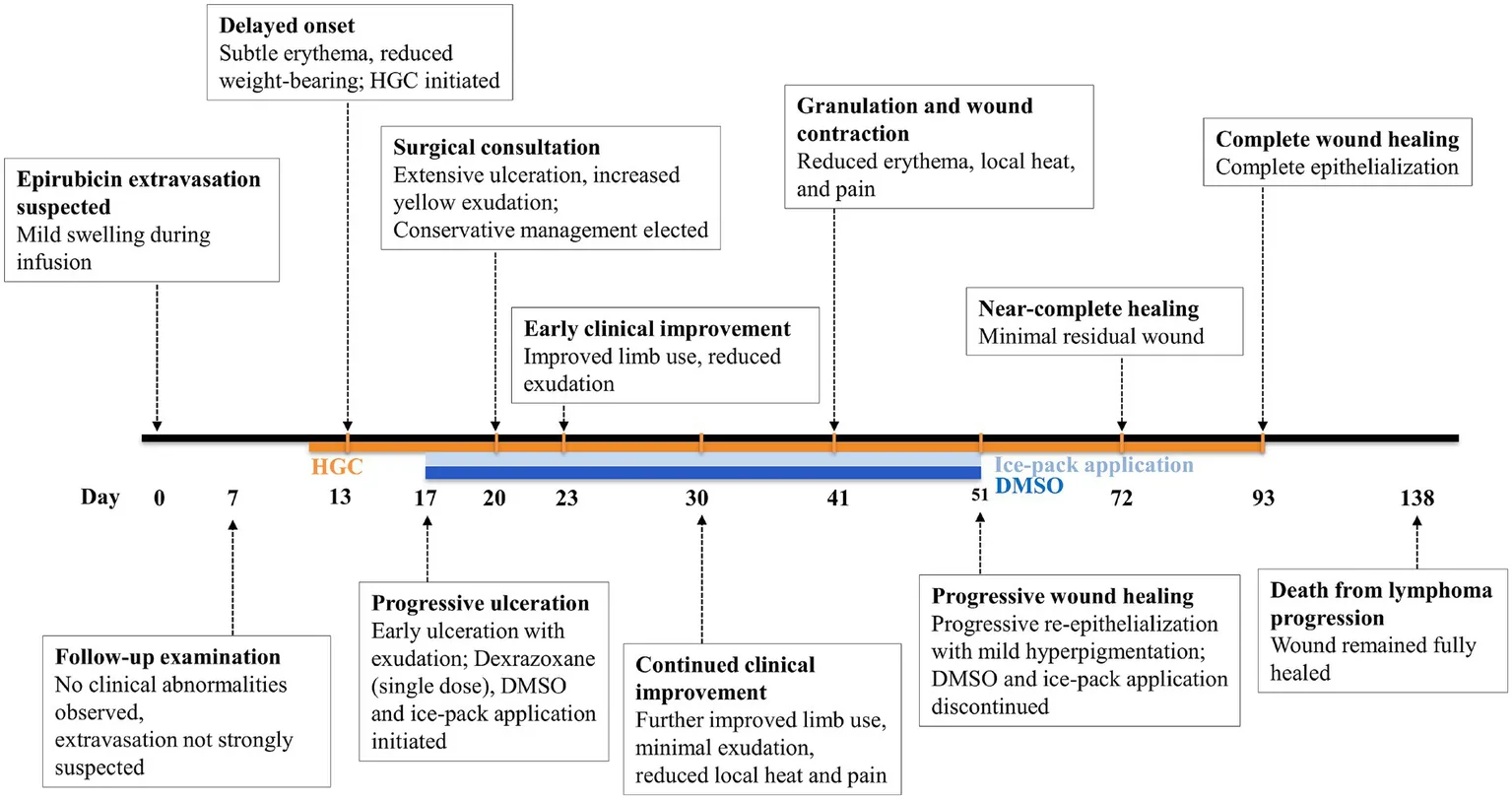
Timeline of the clinical course and treatment interventions. Major clinical findings and duration of adjunctive therapies from the day of the suspected extravasation event through complete wound healing. Days indicate the number of days after the suspected extravasation event. Orange tick marks denote the days of HGC application. HGC, hyperbaric gaseous cryotherapy; DMSO, dimethyl sulfoxide.

Administration was immediately discontinued, and aspiration was attempted to withdraw any residual drug. Catheter function remained intact, with unobstructed saline flow and persistent blood return. No pain response was observed during epirubicin administration before the swelling was noted. These findings made extravasation appear less likely at the time, although preserved blood return does not exclude the possibility. The catheter was subsequently removed, and the remaining dose was administered through a new catheter placed in the right lateral saphenous vein. Based on the initial clinical assessment, dexrazoxane and DMSO were not initiated. However, the owner was informed of the possibility and potential severity of extravasation. Ice-pack application several times daily was recommended, and the owner was instructed to return promptly if swelling, erythema, pain, ulceration, or other skin changes developed.

At the 7-day follow-up, the owner reported no abnormalities, and physical examination findings were unremarkable. Ice packs had been used only intermittently at home because no observable local changes had developed. However, 13 days after the suspected event, subtle erythema and reduced weight-bearing of the left forelimb developed at the same site. Given the clinical history, lesion location, absence of subsequent catheterization or intravenous drug administration at that site, and delayed clinical course consistent with reported cases of anthracycline extravasation, the lesion was considered most consistent with epirubicin extravasation-associated tissue injury (3, 18). Because the lesion was recognized well beyond the recommended treatment window for dexrazoxane and DMSO and clinical signs were relatively mild at that stage, these treatments were not initiated. Instead, adjunctive HGC (Cryo One; Hanil Medipia Co., Ltd., Seoul, South Korea) was applied twice for symptomatic management. Two days later, mild erythema with early superficial skin erosion developed (Figure 2A). By day 17, erythema had worsened, and early ulceration with exudation was observed, alongside increasing pain (Figure 2B). A single dose of dexrazoxane (300 mg/m^2^, IV) was administered. Topical 70% DMSO gel containing 30% *aloe vera* (Nature’s Gift; DMSO, LLC, Ghent, KY, United States) together with ice-pack application were prescribed for home management. By day 20, the lesion had progressed to more extensive ulceration, increased yellow exudation, and crusting (Figure 2C). Physical examination revealed marked hyperemia of the affected limb, with the surrounding tissue warm and moist on palpation. The patient refused weight-bearing on the affected limb while standing and sitting, and even gentle manipulation near the lesion elicited vocalization.

**Figure 2:**
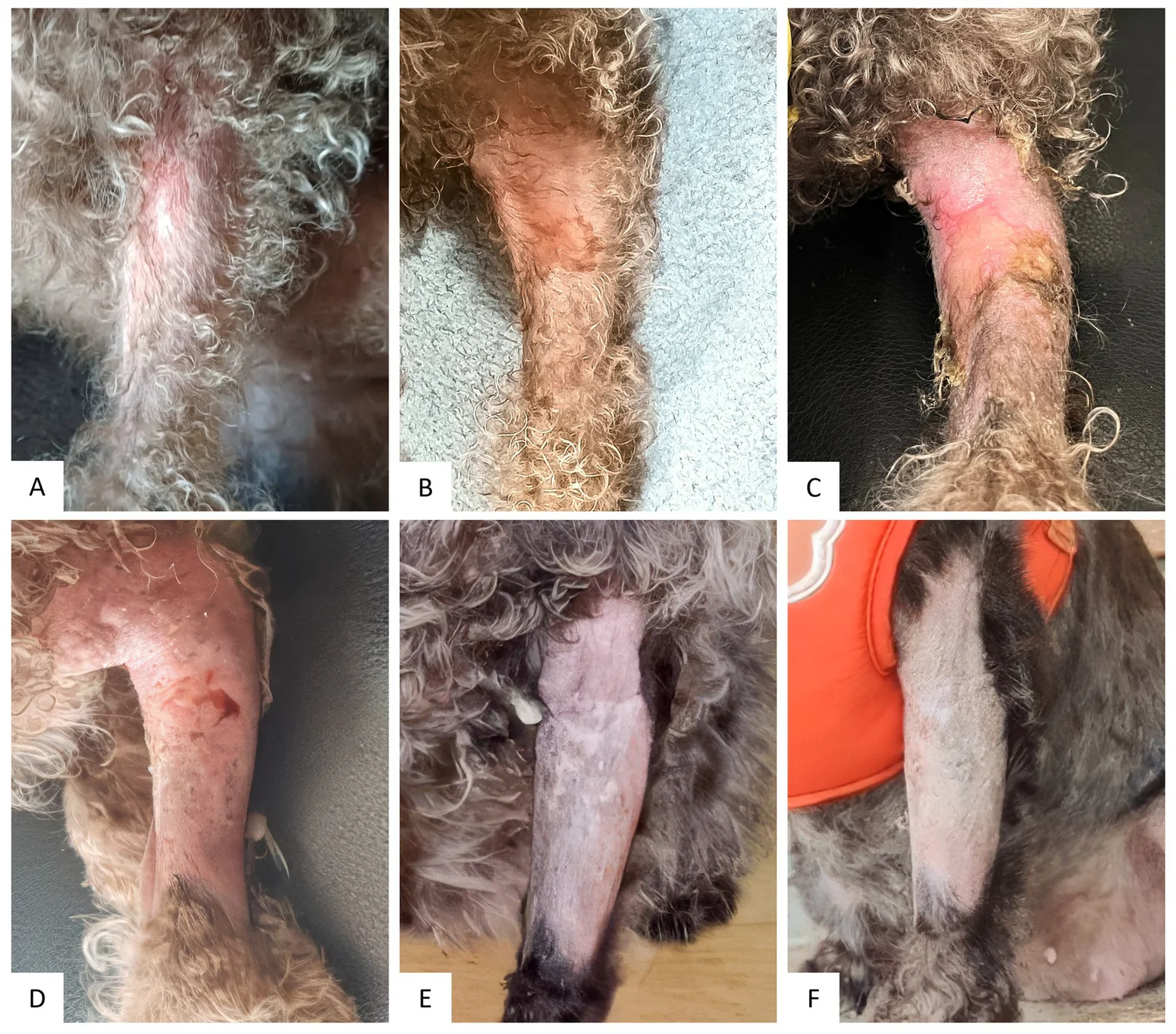
Clinical course of delayed epirubicin extravasation. Days indicate the number of days after the suspected extravasation event. **(A)** Day 15: Mild erythema with early superficial skin erosion. **(B)** Day 17: Early ulceration with exudation and worsening erythema. **(C)** Day 20: Extensive ulceration with yellow exudation and crusting. **(D)** Day 41: Granulation tissue with progressive wound contraction and further reduction in erythema. **(E)** Day 51: Progressive re-epithelialization with mild post-inflammatory hyperpigmentation. **(F)** Day 93: Complete epithelialization with partial hair regrowth.

Given the extent of ulceration and progressive tissue injury, surgical debridement was considered following surgical consultation. However, the patient’s poor response to antineoplastic treatment was an important consideration in determining whether to proceed with surgery, as complete remission had never been achieved during the initial L-CHOP cycle and a subsequent trial of lomustine as rescue therapy also resulted in a limited response. Intermittent hematologic toxicity requiring treatment delays further complicated the treatment course. Considering the limited prognosis associated with this clinical course and the owner’s preference to prioritize quality of life, conservative management was pursued.

HGC was selected as the principal adjunctive modality within the conservative management strategy, based on prior clinical experience in wound management and pain control. On day 20, two HGC sessions were performed approximately 3–4 h apart. Each session consisted of two 1.5-min applications separated by a 30-s rest interval. The nozzle was maintained approximately 2–3 cm from the wound surface and moved continuously in a circular pattern over the ulcerated area and surrounding skin to avoid prolonged cooling of a single area. HGC was periodically passed over the dorsum of the operator’s hand to subjectively assess the degree of cooling. Mild discomfort was observed during application to the lesion. Overall, the procedure was well tolerated with manual restraint and did not require sedation. No new local tissue injury or other adverse changes were observed after HGC application. Immediately following the first HGC session, local heat appeared subjectively reduced, and the patient appeared more comfortable. Behaviors suggestive of discomfort, including repeated attempts to lick the lesion and resistance to handling of the affected limb, were reduced after treatment. As surgical intervention was not pursued and few treatment options remained at this stage, dexrazoxane, DMSO, and ice-pack application were incorporated alongside HGC as part of the conservative management strategy. These interventions were used to maximize supportive care despite the elapsed therapeutic window. Home management consisted of topical DMSO applied three times daily together with twice-daily ice-pack application, with variable owner compliance. Topical DMSO was allowed to air dry without an overlying dressing, as previously described for anthracycline extravasation (10, 19–21). This approach also facilitated repeated ice-pack application to the affected area. Given the location of the lesion at the left cephalic catheterization site, routine contact with the ground or other surfaces was considered limited, and an Elizabethan collar was used to prevent licking and self-trauma.

Subsequent HGC sessions were performed on days 23, 30, 41, 51, 72, and 93, using the same protocol as on day 20. In the absence of an established HGC treatment schedule for anthracycline extravasation, treatment intervals were determined clinically according to wound progression and follow-up availability, with longer intervals used as the lesion improved. On day 23, the patient showed improved limb use but continued to intermittently avoid weight-bearing on the affected limb. Yellow exudation had decreased compared with day 20, though local warmth and moisture remained. By day 30, limb use had further improved, and exudation was minimal. Local heat, moisture, and pain persisted but were less pronounced than at the previous assessment. Pain-related signs progressively decreased following conservative multimodal management, and no systemic analgesic treatment was initiated during the subsequent treatment course. By day 41, erythema and pain had further reduced, and granulation tissue with progressive wound contraction was evident (Figure 2D). Advancing re-epithelialization with mild post-inflammatory hyperpigmentation was observed by day 51 (Figure 2E). Topical DMSO and ice-pack applications were discontinued at this time because local warmth and inflammation had substantially decreased. By day 72, the wound bed was largely covered with newly formed epithelium, with only a small residual area of ulceration remaining. Complete epithelialization and partial hair regrowth were observed by day 93, at which time HGC was discontinued (Figure 2F).

During conservative management of the extravasation lesion, the L-CHOP protocol was continued. Although prednisolone was included in the L-CHOP protocol, it was not administered continuously during this period but was initiated on day 110 as lymphoma treatment transitioned to a palliative approach. By this time, lymphoma had progressed with suspected pulmonary involvement based on pulmonary nodules identified on thoracic radiographs. The extravasation lesion, however, resolved completely without surgical intervention, and the owner expressed satisfaction with this outcome. Complete wound healing was maintained for 45 days until the patient died of lymphoma progression on day 138.

## 3 Discussion

Delayed anthracycline extravasation presents a particular clinical challenge because recommended interventions are most effective when initiated shortly after the extravasation event (10). In the present case, progressive ulceration became evident approximately 17 days after suspected epirubicin extravasation, well beyond the recommended treatment window for dexrazoxane, DMSO, and ice-pack application.

This delayed progression is consistent with the known biological behavior of anthracyclines within affected tissues. Anthracyclines intercalate DNA and inhibit topoisomerase II, resulting in persistent double-stranded DNA breaks (22). Due to their strong affinity for DNA, anthracyclines remain tightly bound within the cell until cell death, at which point they are released and taken up by adjacent viable cells. This self-propagating cycle of cellular uptake, death, and re-release allows prolonged intracellular retention and redistribution within affected tissues, providing a biological explanation for delayed clinical progression and ulcer formation (21). Consistent with this mechanism, persistent epirubicin concentrations have been detected within affected tissues weeks after extravasation despite treatment with DMSO and cold compresses, with progressive necrosis ultimately requiring surgical excision (21). In addition, anthracyclines undergo redox cycling, generating reactive oxygen species (ROS) that contribute to oxidative stress, endothelial damage, microvascular thrombosis, and progressive tissue necrosis (23). Experimental models have confirmed that tissue injury may continue to evolve for days after the initial extravasation event, progressing from edema and hyperemia to ulceration and necrosis (24). These mechanisms collectively explain why lesions that initially appear mild may continue to progress over subsequent days or weeks, as observed in the present case (21, 24). Similarly, delayed ulceration has also been reported following apparent clinical resolution of doxorubicin extravasation, suggesting that clinically silent tissue injury may persist long after initial improvement (3).

Given the severity and delayed presentation of the lesion, surgical debridement was initially considered. According to the Veterinary Cooperative Oncology Group Common Terminology Criteria for Adverse Events (VCOG-CTCAE v2), the presence of ulceration is consistent with a Grade 3 extravasation injury, defined as severe local tissue damage for which surgical intervention is indicated (25). The clinical progression observed in the present case resembled that reported in a human patient with epirubicin extravasation, in which initially mild swelling and pain progressed over approximately 20 days to extensive tissue necrosis requiring surgical debridement and reconstructive surgery (18). When surgical management is indicated, earlier surgical intervention has been associated with more favorable recovery, whereas delayed surgery after established necrosis resulted in prolonged hospitalization and poorer functional outcomes (26). Consistent with these findings, limb amputation has been reported in dogs with severe doxorubicin extravasation (3, 15). Taken together, these reports suggest that surgical intervention is commonly considered once progressive ulceration and tissue necrosis become established. However, the decision to pursue surgery may also depend on the individual characteristics of the lesion and the patient’s overall clinical condition. In the present case, the anticipated benefits of surgical intervention were weighed against the patient’s limited prognosis considering the poor response to antineoplastic treatment, as well as the owner’s preference to prioritize quality of life. Under these circumstances, conservative wound management was considered more appropriate.

As surgery was not pursued and no established treatment existed for delayed anthracycline extravasation, available interventions were used as part of conservative management despite limited evidence supporting their use beyond the recommended treatment window. Conservative management primarily consists of dexrazoxane, topical DMSO, and cold compresses, all of which depend on prompt initiation (1, 10). Among these interventions, dexrazoxane is currently the only FDA-approved agent for anthracycline extravasation in human medicine (12). The recommended administration window for dexrazoxane is within 6 h of the extravasation event, with additional doses at 24 and 48 h (1, 13). Topical DMSO is recommended for early initiation and is typically applied every 6–8 h for 7–14 days (19, 27). Immediate cooling with cold compresses is recommended as first-line emergency management following extravasation (1, 10, 20). Local tissue cooling reduces anthracycline uptake in keratinocytes, decreases membrane transport, and induces vasoconstriction, limiting further tissue distribution of the drug (28). In the present case, however, dexrazoxane, DMSO, and ice-pack application were initiated after clinically significant tissue injury had developed on day 17, well beyond the recommended therapeutic window. Their individual contributions to wound resolution therefore remain uncertain. Evidence supporting dexrazoxane administration at a markedly delayed stage such as this is lacking. Given the marked progression of the lesion and the absence of an established treatment protocol for delayed presentations, dexrazoxane was administered as a single salvage dose in an attempt to limit further tissue injury. As the benefit of repeated administration at this stage has not been demonstrated, the single salvage dose was not extended to the conventional three-day regimen (1, 13). These time-dependent limitations were also observed in a veterinary report where three dogs treated with dexrazoxane within 2 h of doxorubicin extravasation recovered without surgery, whereas the only dog treated after a 48-h delay developed extensive tissue necrosis requiring limb amputation despite dexrazoxane, DMSO, and cold compresses (15). Evidence for DMSO initiation at a markedly delayed stage also remains limited, although one prospective human study included a single patient who began topical DMSO 7 days after anthracycline extravasation, without separately reporting the clinical course of this patient (27). Given the absence of an established treatment protocol and limited treatment options for delayed ulcerative extravasation, DMSO was incorporated as one component of multimodal conservative management rather than as an established treatment for this setting.

Among these approaches, HGC was the primary modality distinguishing the present case from conventional management. HGC provides rapid and localized tissue cooling through a non-contact spray of carbon dioxide microcrystals (29). Compared with conventional cold-pack application, HGC has been shown in dogs to produce more rapid tissue cooling and greater temperature reduction at deeper tissue levels (30). This characteristic was considered potentially relevant because anthracycline-associated tissue injury may extend beyond the superficial dermis into deeper soft tissues through ongoing drug redistribution and secondary inflammatory processes (21). Application of HGC for anthracycline extravasation has not been previously reported in veterinary medicine. In the present case, HGC was selected following surgical consultation as an adjunctive modality within conservative wound management, based on prior clinical experience in wound management and pain control. Previous studies have demonstrated that HGC produces rapid reductions in tissue temperature accompanied by vasoconstriction (30, 31). These physiological effects may reduce edema, modulate local inflammation, and limit secondary tissue injury (28, 30, 32). In the acute setting, rapid tissue cooling may also restrict blood flow and limit further distribution of cytotoxic agents within affected tissues; however, the relevance of this mechanism at the delayed stage observed in the present case is uncertain (28). Pain management was also an important treatment consideration given the marked discomfort associated with progressive ulceration. Cryotherapy has been shown to reduce nerve conduction velocity and modulate nociceptor activity, resulting in reduced pain perception (33). Hyperbaric CO₂ cryotherapy has also been investigated as an analgesic modality, although supporting clinical evidence remains limited (34). In this case, subjective reductions in local heat and pain-related behaviors were observed immediately following HGC sessions, suggesting a potential role for HGC in symptomatic management.

The low clinical incidence of chemotherapy extravasation and the limited availability of reproducible experimental models in veterinary medicine contribute to the scarcity of evidence needed to establish standardized management protocols for delayed anthracycline extravasation. To the authors’ knowledge, this is the first veterinary report describing complete resolution of delayed anthracycline extravasation-associated ulceration through conservative management incorporating adjunctive HGC in a presentation in which surgical intervention would typically be considered. As a single-case report, the individual contribution of HGC cannot be separated from concurrent interventions, and these findings should be interpreted in light of this limitation. The lack of objective tissue temperature monitoring during and after HGC application represents an additional limitation, as changes in tissue temperature were not quantitatively assessed. Although wound progression and healing were documented through serial photographs, wound measurements were not obtained, and changes in pain and limb use were assessed subjectively through physical examination and owner observations without standardized scoring systems. Nonetheless, the favorable clinical course observed in this patient adds to the limited veterinary evidence regarding conservative management of delayed anthracycline extravasation and provides a clinical basis for further investigation of HGC as an adjunctive modality in delayed anthracycline extravasation.

## 4 Conclusion

This case demonstrates that complete resolution of delayed epirubicin extravasation-associated ulceration may be achievable through conservative treatment, even in presentations where surgery would typically be a treatment option. Although surgical management remains an important consideration once progressive ulceration or necrosis is established, this report suggests that in selected patients, particularly when surgery is not feasible or a conservative approach is preferred, surgery-informed conservative management incorporating adjunctive modalities such as HGC may be considered. These findings support further development of evidence-based conservative management strategies for delayed anthracycline extravasation in veterinary patients.

## Data Availability

The original contributions presented in the study are included in the article/supplementary material, further inquiries can be directed to the corresponding author.
